# An Inverse Relation between Hyperglycemia and Skeletal Muscle Mass Predicted by Using a Machine Learning Approach in Middle-Aged and Older Adults in Large Cohorts

**DOI:** 10.3390/jcm10102133

**Published:** 2021-05-14

**Authors:** Xuangao Wu, Sunmin Park

**Affiliations:** 1 Department of Bio-Convergence System, Hoseo University, Asan 31499, Korea; 20195714@vision.hoseo.edu; 2 Obesity/Diabetes Research Center, Department of Food and Nutrition, Hoseo University, Asan 31499, Korea

**Keywords:** skeletal muscle mass, fat mass, machine learning, grip strength, prediction model, platelet, C-reactive protein

## Abstract

Background: Skeletal muscle mass (SMM) and fat mass (FM) are essentially required for health and quality of life in older adults. Objective: To generate the best SMM and FM prediction models using machine learning models incorporating socioeconomic, lifestyle, and biochemical parameters and the urban hospital-based Ansan/Ansung cohort, and to determine relations between SMM and FM and metabolic syndrome and its components in this cohort. Methods: SMM and FM data measured using an Inbody 4.0 unit in 90% of Ansan/Ansung cohort participants were used to train seven machine learning algorithms. The ten most essential predictors from 1411 variables were selected by: (1) Manually filtering out 48 variables, (2) generating best models by random grid mode in a training set, and (3) comparing the accuracy of the models in a test set. The seven trained models’ accuracy was evaluated using mean-square errors (MSE), mean absolute errors (MAE), and R² values in 10% of the test set. SMM and FM of the 31,025 participants in the Ansan/Ansung cohort were predicted using the best prediction models (XGBoost for SMM and artificial neural network for FM). Metabolic syndrome and its components were compared between four groups categorized by 50 percentiles of predicted SMM and FM values in the cohort. Results: The best prediction models for SMM and FM were constructed using XGBoost (R2 = 0.82) and artificial neural network (ANN; R2 = 0.89) algorithms, respectively; both models had a low MSE. Serum platelet concentrations and GFR were identified as new biomarkers of SMM, and serum platelet and bilirubin concentrations were found to predict FM. Predicted SMM and FM values were significantly and positively correlated with grip strength (*r* = 0.726) and BMI (*r* = 0.915, *p* < 0.05), respectively. Grip strengths in the high-SMM groups of both genders were significantly higher than in low-SMM groups (*p* < 0.05), and blood glucose and hemoglobin A1c in high-FM groups were higher than in low-FM groups for both genders (*p* < 0.05). Conclusion: The models generated by XGBoost and ANN algorithms exhibited good accuracy for estimating SMM and FM, respectively. The prediction models take into account the actual clinical use since they included a small number of required features, and the features can be obtained in outpatients. SMM and FM predicted using the two models well represented the risk of low SMM and high fat in a clinical setting.

## 1. Introduction

Societal aging is a global phenomenon, and in Korea, people aged over 65 years constituted 10.7% of the population in 2010, 12.7% in 2014, and 14.3% in 2020. Sarcopenia is characterized by a decline in skeletal muscle mass (SMM) and reduced age-related muscle strength [1]. According to reports, SMM and muscle strength begin to reduce after middle age at an annual rate of 1–2% per year after age 50 [2].

SMM loss increases the risk of falls, comorbidities, and premature death [3]. Maintaining SMM plays a crucial role in promoting quality of life and reducing mortality and morbidity in older adults [4]. SMM plays an essential role not only in daily activities but also in many metabolic pathways. SMM is an essential part of insulin stimulation and acts to maintain glucose homeostasis [3] and is also involved in fatty acid metabolism. Disorders of SMM metabolism lead to insulin resistance, metabolic syndrome, and obesity [3]. In older adults, SMM reduction is linked to increases in fat mass [5]. Furthermore, the prevalence of obesity is increasing rapidly [6], and obesity increases the risk of various metabolic diseases, such as diabetes and cardiovascular disease [6]. In addition, obesity and SMM loss can act synergistically to cause more severe health problems [7,8].

The potential mechanisms responsible for SMM reduction involve changes in anabolic hormonal signaling, protein oxidation, oxidative stress, inflammation, metabolic stress, and neuromuscular junction degeneration, and these processes replace muscle fiber with fats [9,10]. SMM reduction and obesity have many overlapping causes, and it is speculated that the two are closely related and exacerbate each other [7,8]. Researchers have explored the unmodifiable and modifiable risk factors of SMM and FM over the past few decades. However, no research study has investigated the relative importance of a series of unmodifiable and modifiable risk factors. The random forest model of the machine learning approach can find potential biomarkers among many candidates and rank these by relative importance, thus this model has been applied in many studies [11,12,13].

SMM and FM can be estimated by bioelectric impedance (BIA), dual-energy X-Ray absorptiometry (DXA), magnetic resonance imaging (MRI), and computed tomography (CT). However, all of these techniques have limitations in measured SMM and FM accuracy [14]. SMM and FM have been measured in a small number of cohort studies. In KoGES, SMM and FM were measured by BIA only in the Ansan/Ansung cohort (*n* = 8842) but not the city hospital cohort (*n* = 56,486). However, since the Ansan/Ansung cohort was relatively small, it was unsuitable for investigations aimed at identifying novel risk regulators of SMM or FM using association methods. Nonetheless, best prediction algorithms based on integrating the relative importance of risk factors from the Ansan/Ansung cohort using a machine learning approach can be used to predict SMM and FM [12,15]. We hypothesized that the best SMM and FM prediction models could be generated from the Ansan/Ansung cohort using the machine learning approach, and the metabolic characteristics and grip strength were different according to the predicted SMM and FM in the urban hospital-based cohort. We aimed to examine this hypothesis in adults aged > 40 years in two KoGES cohorts.

## 2. Methods

### 2.1. Participants

KoGES included the Ansan/Ansung cohort and the urban hospital-based cohort. Participants were aged 51.97 ± 8.85 and residents of Ansan City (a large city area) or Ansung City (a small city area) from 2001 to 2007. In Ansan/Ansung cohort, body compositions, including SMM and FM, were measured using Inbody 4.0 (Inbody, Seoul, South Korea). After excluding participants lacking SMM and FM data, 6657 participants (3216 men and 3441 women) were included in the present study, and 1411 variables were used to build predictive models of SMM and FM. Ansan/Ansung cohort was used to generate the best prediction model for SMM and FM using a machine learning approach.

The urban hospital-based cohort is another KoGES cohort conducted from 2004 to 2013 and had 53,843 participants, and the predictive model was applied to predict SMM and FM of the participants in this cohort. However, body compositions, including SMM and FM, were not collected. After excluding participants with missing data for predictive model parameters, 31,025 participants (including 10,370 men and 20,655 women) aged 52.23 ± 8.1 were included in the present study. The age distribution of Ansan/Ansung and urban hospital-based cohorts were similar (Supplementary Figure S1A,B).

The study protocol was approved by the Institutional Review Board of the Korea Centers for Disease Control and Prevention (KBP-2015-055) and Hoseo University (1041231-150811-HR-034-01). Written informed consent was obtained from all participants.

### 2.2. Experiment Design

**Figure 1:**
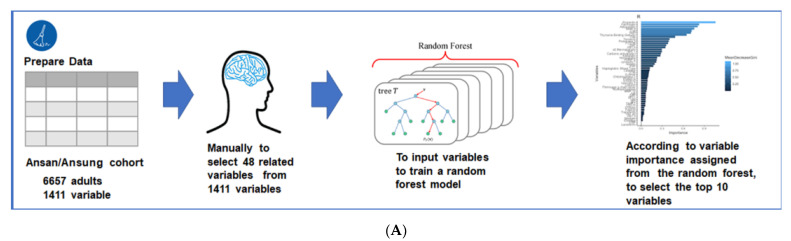

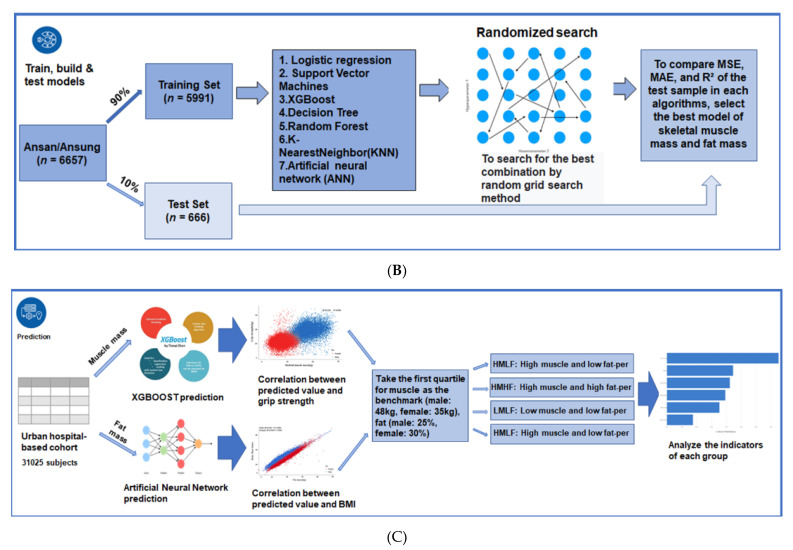
Experimental flowchart. (**A**) Flow to filter features for SMM and FM in Ansan/Ansung cohort. (**B**) Flow to generate the best model by a random grid model in the training set and checking the error in the test set. (**C**) Flow to analyze clinical characteristics of the participants with high or low skeletal muscle mass (SMM) and fat mass (FM) of the urban hospital-based cohort.

The present study was conducted in three parts (Figure 1). First, the sample sizes of the training and testing sets were determined using the G-power test in the criteria of effect size, power, and significance level at 0.1, 0.01, and 0.99, respectively. A sample size of 656 was found to be suitable. Thus, the testing set was defined as 10% of the Ansan/Ansung cohort (*n* = 666). We manually excluded participants with missing values and duplicates from the 1411 in the Ansan/Ansung cohort. Forty-eight variables were used to train a random forest algorithm to predict SMM and rated in order of importance. In order to reduce the number of variables required to predict SMM using the machine learning approach, we removed the variables one by one from low to high importance and iteratively trained the random forest model until the mean square errors (MSEs) of the model did not decrease significantly after eliminating one variable each. Finally, when the top 10 variables had been identified (Figure 1A), we applied the same screening method to the prediction of FM.

The second step was to train seven algorithms using the selected ten variables. The seven machine learning algorithms used were linear regression, support vector machines (SVM), XGBoost, decision tree, random forest, K-nearest neighbor (KNN), and artificial neural network (ANN). The best combination of variables was selected using a random grid search method for each algorithm, and each trained algorithm was used to calculate SMM and FM in the test set. The best algorithm was selected by comparing MSEs, mean absolute errors (MAEs), and R² values of SMM and FM predicted by each algorithm in the test set.

The third step involved predicting the SMMs and FMs of participants in the urban hospital-based cohort using the selected best models for SMM and FM. Clinical characteristics of the participants with a high or low SMM or FM in a city hospital-based cohort are provided in Figure 1B.

### 2.3. Training for SMM and FM Prediction Model

Participants in the Ansan/Ansung cohort were randomly divided 90%:10% to a training set or a test set. The mean age in the SMM training set cohort was 51.38 ± 8.9, and the mean age in the test set cohort was 51.17 ± 8.9 (Supplementary Figure S1C). For FM, the corresponding mean ages were 51.39 ± 8.91 and 51.42 ± 8.72, respectively (Supplementary Figure S1D). The train-test-split package of sklearn was used to train the seven algorithms. In order to find the optimal hyperparameter settings of the models, a random grid search method was used when training each model [16]. A hyperparameter range was set for each model, and the best combination of ten variables was generated from 300 variable combinations within the assigned hyperparameter range. Hyperparameter combinations with the best performances were used as best models. The random forest and XGBoost algorithms returned the best combinations during these processes, each containing ten important variables for SMM and FM. The linear regression model was used to generate correlations between included variables.

### 2.4. Verifying the Predictive Models

The trained models’ accuracies were evaluated using the remaining 10% of the Ansan/Ansung cohort as a test set by calculating mean-square errors (MSE), mean absolute errors (MAE), and R² values of the seven trained models. We also evaluated absolute errors in three SMM and FM ranges by quintiles (Q1, Q2–Q4, and Q5) to check accuracy differences according to the ranges of SMMs and FMs. The optimal SMM prediction model was XGBoost, and its optimal hyperparameters were as follows—84 of trees and 2 of max depth. The optimal FM prediction model was ANN, and its optimal hyperparameter was as follows—10 artificial neurons in the input layer, six artificial neurons in a hidden layer, “relu” of the activation function, and one artificial neuron in the output layer.

### 2.5. Predictions of SMM and FM in the Urban Hospital-Based Cohort Using the Predictive Algorithm Models

We used the two trained models by XGBoost and ANN algorithm to predict SMM and FM, respectively, in the urban hospital-based cohort and investigated the relationships between SMM and grip strength and between FM and BMI. We dichotomized subjects in the urban hospital-based cohort into 4 SMM/FM groups using SMM first quartile cutoffs of 48 kg for men and 35 kg for women and FM cutoffs of 25% for men and 30% FM for women. Accordingly, the four groups were: the HMLF group (high SSM and low FM); the HMHF group (high SSM and high FM), the LMLF group (low SSM and low FM), and the LMHF group (low SSM and high FM). Adjusted means and standard errors were determined for metabolic syndrome component and lifestyle variables after adjusting for age, gender, residence area, education, income, and BMI.

### 2.6. Statistical Analysis

The normality test of each continuous variable was determined, and the variables showed normal distribution. The significances of differences between variables for men and women were determined using the two-sample t-test in the Ansan/Ansung and urban hospital-based cohorts. Linear relationships were analyzed using Pearson’s correlation coefficients. Adjusted means and standard errors were calculated after adjusting for age, genders, residence area, education, income status, and BMI in the four groups (described above) of the urban hospital-based cohort. One-way ANOVA was used to determine the significances of intergroup differences. The analysis was performed using SPSS version 20 (IBM, Chicago, IL, USA), and statistical significance was accepted for *p*-values < 0.05. Results are presented as means ± standard deviations.

## 3. Results

### 3.1. Metabolic Characteristics of the Ansan/Ansung and Urban Hospital-Based Cohorts

The mean subject age was ~51 in the Ansan/Ansung cohort and 54 in the urban hospital-based cohort (Table 1). BMI and waist and hip circumferences were similar in both cohorts. SMM was 37.7 ± 4.5 kg for men and 28.4 ± 3.2 kg for women in the Ansan/Ansung cohort, but it was not provided in the urban hospital-based cohort (Table 1). Grip strength was not measured in the Ansan/Ansung cohort but was higher for men than women in the urban hospital-based cohort (Table 1).

Metabolic parameters, including glucose and lipid levels, inflammation, and blood pressure, were better in the urban hospital-based cohort than in the Ansan/Ansung cohort, and with the exceptions of serum total cholesterol and platelet concentrations, which were better in women than men in both cohorts (Table 1). Energy intakes and proportions of carbohydrates, protein, and fats consumed were similar in the two cohorts. Women had higher energy intakes based on estimated energy requirements and carbohydrate intakes than men but had lower fat intakes than men (Table 1). Anthropometric and metabolic parameters showed similar gender relationships in both cohorts.

### 3.2. Relative Importance of Parameters in the Random Forest and XGBoost Prediction Models

**Figure 2:**
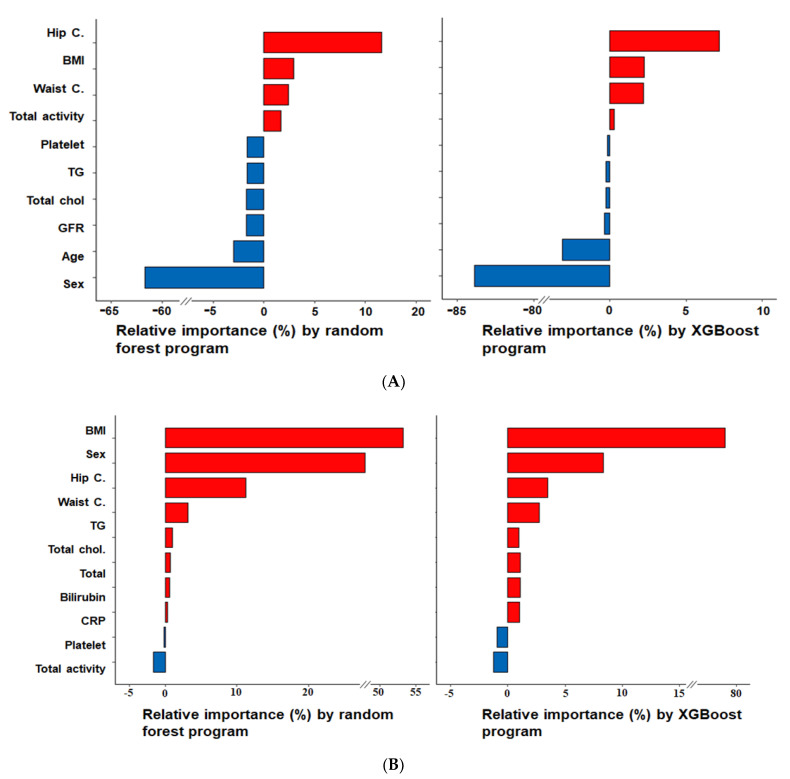
The relative importance of variables for predicting SMM and FM, as determined by the random forest and XGBoost algorithm. (**A**) Skeletal muscle mass (SMM); (**B**) fat mass (FM). BMI, body mass index; C., circumferences; GFR, glomerular filtration rate; Total chol., serum total cholesterol concentrations; TG, serum triglyceride concentrations.

Random forest and XGBoost algorithms in the seven trained ones returned the importance of features (Figure 2A,B). Of these features, hip circumferences and BMI were the most important predictors for SMM, whereas BMI and gender were for FM. According to the linear regression analysis, hip circumference, BMI, waist circumference, and total activity were positively associated with SMM, whereas age, glomerular filtration rate (GFR), female gender, and serum concentrations of total cholesterol, triglyceride, and platelets exhibited negative associations (Figure 2A). FM was predicted using the method used for SMM. Parameters with high relative importance were BMI, gender, hip circumference, and waist circumference in both algorithms, such as random forest and XGBoost. BMI, female, hip circumference, waist circumference, and serum triglyceride, total cholesterol, total bilirubin, and CRP concentration positively influenced FM prediction. However, serum platelet concentrations and total activity were negatively affected by physical activity serum platelet concentration (Figure 2B).

### 3.3. Accuracies of the Predictive Models for SMM and FM Using XGBoost and ANN Algorithm in the Test Set 

The MSE and MAE values of the XGBoost SMM predictive models were the smallest, and R² was close to 1, indicating the model using the XGBoost algorithm exhibited strong relationships. The model’s MSE and MAE values for FM prediction using the ANN algorithm were the smallest, and its R² was also close to 1 (Table 2).

**Figure 3:**
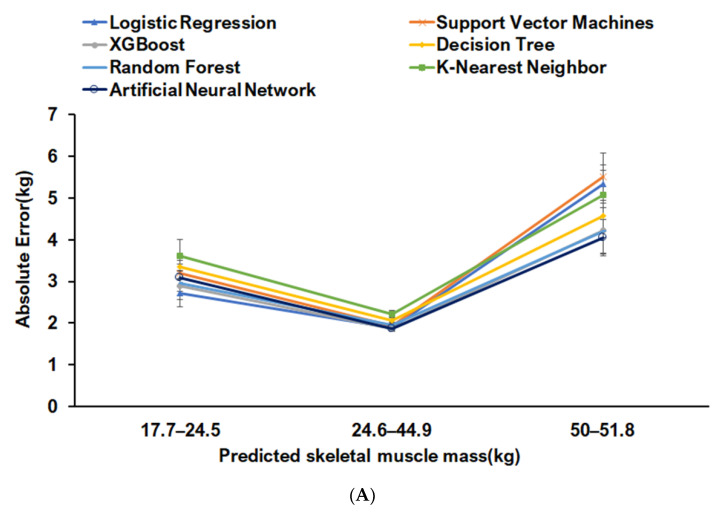

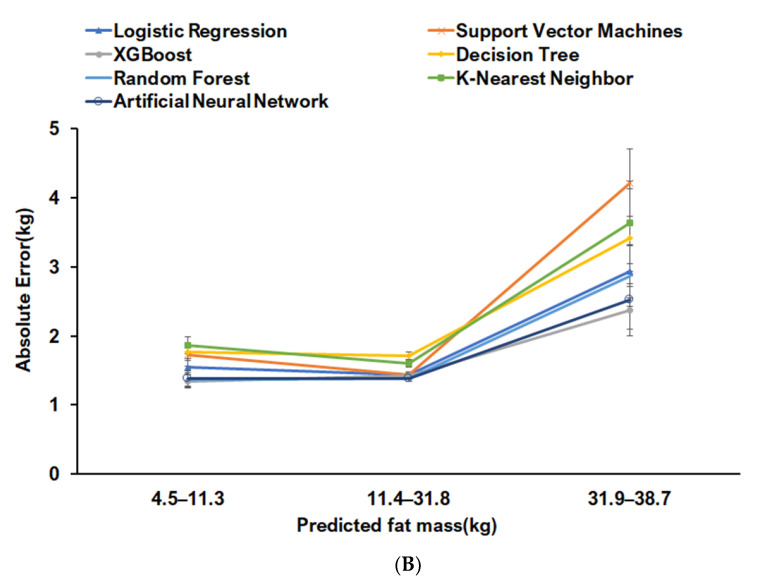
Absolute errors of SMM prediction with ranges in the test set for the seven machine learning algorithms. (**A**) Skeletal muscle mass (SMM); (**B**) fat mass (FM). Absolute errors were calculated by subtracting actual values from predicted values. The SMM and FM were divided into quintiles and Q1, Q2–Q4, and Q5. The absolute errors of predicted SMM and FM by XGBoost and ANN models were calculated in each of these three ranges.

The random forest prediction model was applied to seven machine learning algorithms (Table 2, Figure 3A,B). Predicted SMM had an absolute error of 7% in the test set. XGBoost was the best predictive model for SMM. Predicted SMM by XGBoost was divided into three groups by 20th and 80th percentiles. Mean square errors were lowest in the 20th–80th group for SMM prediction, and the absolute error was <2%. Mean square errors were higher in the <20th and >80th percentile groups than in the 20th–80th percentile. Linear regression, XGBoost, random forest, and ANN models showed the lowest absolute errors. Since the absolute error of the best prediction model for SMM was highest in the <20th percentile group, it was unsuitable for subjects with a low SMM (Figure 3A).

In the FM predictive models provided by seven machine learning algorithms, the absolute error percentage in overall FM was ~10%, and it was bigger for FM than SMM. The prediction models generated by XGB, random forest, and ANN algorithm had the lowest absolute errors for FM prediction in all FM ranges. The absolute error was ~5% in the 20th–80th percentile range for the ANN model, and the prediction of FM in the > 80th percentile range exhibited a much higher absolute error value (Figure 3B).

### 3.4. Anthropometric and Metabolic Parameters According to Predicted SMM and FM in Men and Women in the Urban Hospital-Based Cohort

Since men and women had significantly different SMM and FM, SMM and FM cutoffs were defined separately for men and women (Table 3 and Table 4). The cutoffs (75th percentiles) of SMM for men and women were 48 and 36 kg, respectively, whereas those of FM were 25% and 30%, respectively. Predicted SMM increased in the order LMLF, LMHF, HMLF, and HMHF, whereas predicted FM reduced HMHF, LMHF, HMLF, and LMLF. As was expected, BMI and waist and hip circumferences were higher in the HMHF, LMHF, HMLF, and LMLH groups, and order was similar to SMM and FM. However, grip strength was highest in the HMLF group and lowest in the LMHF group.

In men, serum glucose and HbA1c concentrations were higher in the high-fat groups, regardless of SMM. Serum triglyceride concentrations reduced in the order LMLF, HMLF, and HMHF = LMHF, whereas serum HDL concentrations increased in the order HMHF, LMHF = HMLF, and LMLF. GFR was higher for a low SMM and a high FM. SMM and waist and hip circumferences in women showed a similar trend to those observed in men. Grip strength was highest in the HMLF group and lowest in the LMHF group. In women, serum concentrations of glucose and HbAlc were higher in the HF than in the LF groups regardless of SMM. GFR exhibited different trends in men and women. In men, GFR was higher for low SMM and FM than for high SMM and FM, but in women, GFR was higher in high SMM and low-FM than for low SMM and high FM.

### 3.5. Linear Relationship between SMM and Grip Strength in the Urban Hospital-Based Cohort

**Figure 4:**
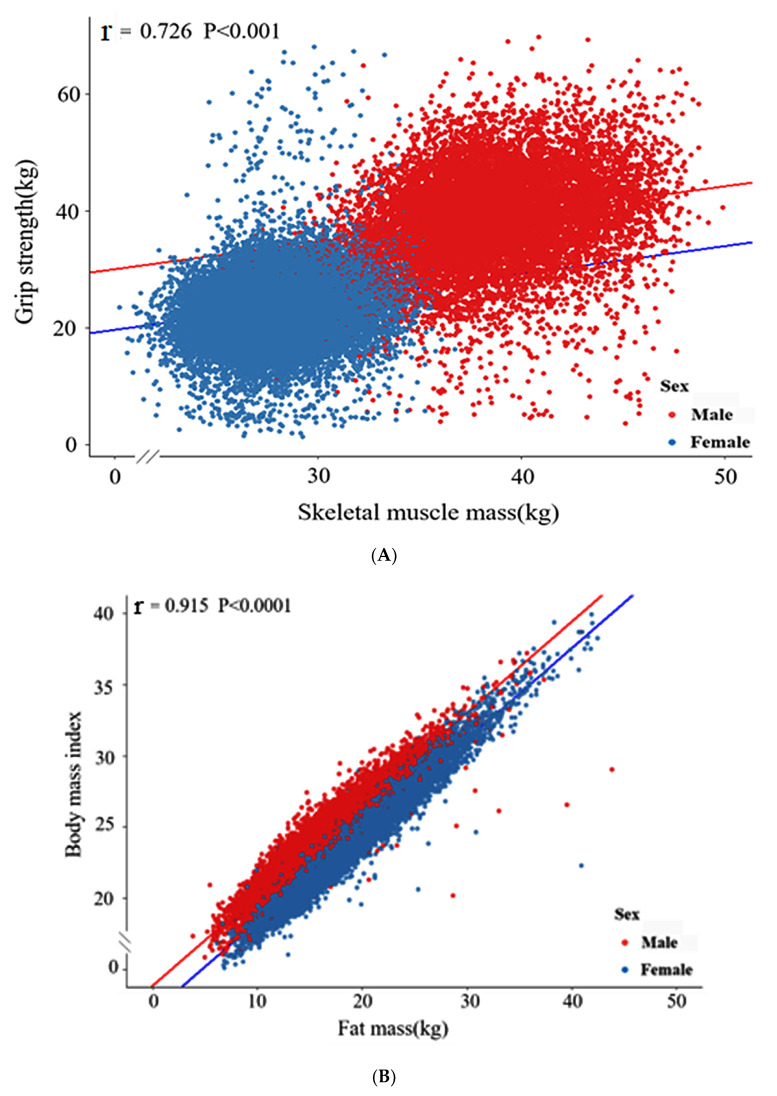
Linear regression analysis. (**A**) The relation between skeletal muscle mass and grip strength; (**B**) The relation between fat mass and body mass index. r, Pearson’s correlation coefficient. The *p*-value for the correlations.

Since grip strength is positively correlated with SMM and negatively associated with sarcopenia [1,2], the XGBoost model’s reliability for predicting SMM was examined with the relationship between grip force and SMM using linear regression analysis. Grip strength and SMM showed a significant linear relationship (*p* < 0.001, Figure 4A) with a Pearson’s correlation coefficient (r) value of 0.726, indicating the strongest correlation. BMI also showed a significant linear relationship with FM in both genders (*p* < 0.001, Figure 4B). The r-value (correlation coefficient) of the correlation between SMM and grip strength in the urban hospital-based cohort was 0.915.

## 4. Discussion

Machine learning has been attracted considerable attention during recent years, and machine learning data processing, clustering, classification, dimensionality reduction, regression, and other functions provide potent data mining tools. Unlike traditional computer algorithms that produce results when commands are executed, machine learning algorithms can construct and train algorithms to process data and obtain results in required forms [3]. In the medical field, machine learning-based predictive algorithms have been developed to analyze metabolic factors related to various diseases [4,5,6], and in the present study, we used machine learning algorithms to create regression models that predict SMM and FM. In men and women, muscle mass accounts for 47–60% of body weight and maintains energy expenditure throughout the body [7]. Therefore, promoting skeletal muscle mass is essential for maintaining the quality of life and extending health expectancy. Women tend to experience more rapid muscle loss than men due to lower testosterone concentrations and muscle renewal rates [8]. In women, estrogen deficiency after menopause reduces bone density [9], muscle mass [10], and increases FM [11], thus SMM and FM vary considerably between genders.

In the importance analysis, the random forest [12] and linear regression [13] showed the importance of variables and their positive and negative correlations, respectively. Random forest and XGBoost algorithm were used to analyze the importance of variables and select the top 10 by importance. In the prediction model for SMM, the variables in descending order of importance were sex, hip circumference, age, BMI, waist circumference, GFR, total activity, and serum concentrations of total cholesterol, triglycerides, and platelets. Previous studies have reported that BMI, sex, hip circumference, waist circumference, and BMI are related to SMM and FM [14,15], and that age is negatively correlated with SMM [16]. GFR levels are low in patients with chronic kidney disease, who usually exhibit SMM loss [17], and a study on patients with chronic diabetic kidney complication reported reductions in lean body mass. These results suggested that appendix skeletal muscle loss is significantly related to GFR decline [17]. Platelet levels reflect the destruction of blood vessels and tissues, as platelets play crucial roles in hemostasis and wound healing [18]. Furthermore, platelet activation plays a central role in accelerating atherothrombosis related to insulin resistance, inflammation, oxidative stress, and endothelial dysfunction [19]. According to the National Health and Nutrition Examination Survey (1999 to 2004), mean platelet volume is strongly associated with the presence of type 2 diabetes [20]. However, the clinical effects of platelets on skeletal are still unclear. In our SMM model, platelet levels and SMM showed a weak negative correlation, which suggests platelets may have different effects on skeletal muscles in the presence of different combinations of insulin resistance, inflammation, and protein synthesis.

In the present study, the FM prediction model using ANN included BMI, sex, hip circumference, waist circumference, total activity, and serum triglyceride, total cholesterol, total bilirubin, CRP, and platelet concentrations. Excessive fat accumulation is distributed in subcutaneous tissues related to disruption of lipid metabolism and inflammation in obese individuals to fatty liver and dyslipidemia [21]. In the present study, the FM prediction model using ANN indicated that total body fat and predicted serum triglyceride and total cholesterol concentrations were positively correlated with FM.

Plasma bilirubin concentration, an index of liver and gallbladder damage, is positively related to hepatic fat accumulation [22], and our FM model showed total bilirubin was weakly and positively correlated with predicted FM.

In the present study, we compared the accuracies of regression models using machine learning algorithms and selected the best prediction models for SMM and FM. Of the seven machine algorithms examined, random forest, XGBoost, and ANN had the lowest MSEs for predicting SMM and FM in the Ansan/Ansung cohort test set. XGBoost and ANN have also been reported to achieve good accuracies, and XGBoost has been shown to enable the identification of biomarkers [23,24]. Grip strength is applied to validate SMM’s prediction because grip strength is generally considered to reflect muscle strength [25]. Because no direct measurements could be used to judge the accuracy of SMM predictions in the urban hospital-based cohort, we examined the correlation between SMM and grip strength and found grip strength and SMM were strongly correlated, which suggested predicted SMMs were reliable. On the other hand, we found predicted FM was linearly and positively correlated with BMI. Thus, it appears that predicted values of SMM and FM in the urban hospital-based cohort are reliable.

We also divided anthropometric parameters into the HMLF, HMHF, LMLF, and LMHF groups to predict SMM and FM in men and women. Both male and female HM groups had higher grip strengths than their counterparts in the LM groups, which is consistent with the results of a previous study regarding the correlation between SMM and grip strength [25]. Serum triglyceride (a biochemical indicator of obesity) levels were higher in men and women in HF groups than in LF groups [21]. Impaired glucose tolerance (IGT) can contribute to an increase in fasting blood glucose concentration, a critical factor of visceral fat accumulation [26], which can lead to obesity and is closely related to the risk of type 2 diabetes [27]. Both men and women in the HF groups had higher blood glucose levels than their counterparts in the LF groups, which might have been related to IGT caused by fat accumulation. HbA1c is an essential indicator of the diagnosis of diabetes and reflects an individual’s blood glucose level 2 to 3 months after testing [28]. As was observed for blood glucose data, men and women in the HF groups had higher HbA1c ratios than those in the LF groups, and women in the LMHF group had the highest mean HbA1c ratio.

We trained multiple prediction models for SMM and FM using Ansan/Ansung data and eventually obtained SMM and FM predictions for urban hospital-based cohorts using the XGBoost and ANN models. The maximum R² values of the SMM and FM models were 0.82 and 0.89, respectively, which is in line with actual application indicators in the medical system. The results of the present study can be applied to clinical setting since the SMM and FM had specific characteristics as follows: (1) The prediction model of SMM and FM included relatively small number of variables, (2) variables included in the prediction algorithm was not fluctuated quickly in a short time, and (3) SMM and FM of the adults fell in the accurate ranges of prediction algorithm [29]. Therefore, these models can quickly and accurately predict SMM and FM values through existing routine test data in a clinical environment. These models can be used for the accurate prediction of SMM and FM at a low cost. Many cohorts lack SMM and FM data, and these models can also be used to predict missing data and conduct research. Furthermore, the predicted SMM and FM of the participants in the urban hospital-based cohort were found to well-represent the clinical characteristics of SMM and FM like grip strength and hyperglycemia. Therefore, the ten variables in the prediction models could be used to be modulated to prevent SMM reduction or FM elevation in a clinical setting.

However, the trained prediction models have several limitations. (1) Since they were trained using Korean data, our results may be unsuitable for applications in European or Hispanic populations. (2) Our study was performed on Koreans ≥40 years old and thus our findings may not be applicable in younger populations. (3) SMM and FM values determined in the Ansan/Ansung study were obtained using the Inbody machine, which is less accurate than DXA. However, we confirmed the applicability of the prediction models in a large urban hospital-based cohort, and the accuracy of predicted values was verified by linear relationships between grip strength and SMM and BMI and FM. (4) Regarding the feature importance analysis, we used simple XGBoost, random forest, and linear regression to explore important variables without knowing the impact of each variable. However, according to Lundberg’s research on SHapley Additive exPlanations (SHAP), the SHAP value can explain how each feature affects machine learning algorithms, providing a new interpretation method for the machine learning black box algorithms [30]. In the future study, SHAP algorithm needs to be used to determine the impact of each variable in the prediction model. GFR and serum platelet concentrations among the ten variables screened by random Forest and XGBoost have also shown to be potential biomarkers for SMM and FM, as shown to the possibility as biomarkers in previous studies [21,26,27,28]. Furthermore, our study shows that Koreans with a high FM have higher fasting blood glucose levels and suggests a higher risk of type 2 diabetes in this population.

## 5. Conclusions

The best prediction models, which included ten variables for SMM and FM, were generated using XGBoost and ANN algorithms, respectively, using the Ansan/Ansung cohort. The SMMs and FMs predicted by the models well represented the risk of low SMM and high FM in this hospital-based cohort. Adults with a low SMM and high FM are at risk of hyperglycemia, and the prediction models included potential biomarkers for SMM, such as serum platelet levels, triglyceride concentrations, and GFR. In contrast, those for FM included serum bilirubin and triglyceride concentrations. The prediction algorithm for SMM and FM can be considered to use in the clinical site since the variables in the algorithm are easy access. The prediction models are of practical significance in clinical settings and the predicted SMM and FM values can convince the patients to modulate their diet and physical exercise plans.

## Figures and Tables

**Table 1:** The characteristics of participants according to the genders in the Ansan/Ansung and urban hospital-based cohorts.

Variables	Ansan/Ansung Cohort		Urban Hospital-Based Cohort	
	Men (3216)	Women (3441)	Men (*n* = 10,370)	Women (*n* = 20,655)
Age (years)	50.6 ± 8.46	51.8 ± 8.88 ***	55.2 ± 8.30	53.1 ± 7.75 ***
Body mass index (kg/m^2^)	24.4 ± 2.86	24.8 ± 3.24 ***	24.5 ± 2.71	23.6 ± 2.98 ***
Waist circumferences (cm)	83.6 ± 7.51	80.6 ± 9.49 ***	85.5 ± 7.49	77.8 ± 8.05 ***
Hip circumferences (cm)	94.4 ± 5.54	94.1 ± 5.96	95.4 ± 5.71	92.8 ± 5.8 ***
Skeletal muscle mass (kg)	37.7 ± 4.51	28.3 ± 3.21 ***	-	-
Fat mass (kg)	15.2 ± 4.81	19.0 ± 5.29 ***	-	-
Grip strength (kg)	-	-	38.5 ± 0.09	23.3 ± 0.04 ***
Serum glucose (mg/dL)	116 ± 16.3	115 ± 19.2	100 ± 22.9	93.8 ± 18.1 ***
Blood HbA1c (%)	5.78 ± 0.02	5.73 ± 0.01	5.78 ± 0.01	5.68 ± 0.005 ***
SBP (mmHg)	75.8 ± 11.4	72.9 ± 11.9 ***	38.5 ± 0.09	23.3 ± 0.04 ***
DBP (mmHg)	91.1 ± 24.4	85.2 ± 20.3 ***	100 ± 22.9	93.8 ± 18.1 ***
Serum triglyceride (mg/dL)	177 ± 118	146 ± 86.5 ***	148 ± 102	114 ± 74.0 ***
Serum HDL (mg/dL)	43.4 ± 9.69	45.9 ± 10.2 ***	50.2 ± 12.3	57.6 ± 13.7 ***
Serum total cholesterol (mg/dL)	194 ± 36.3	192 ± 36.2 **	193 ± 35.9	201 ± 36.3 ***
Serum CRP (mg/dL)	0.23 ± 0.44	0.21 ± 0.41	0.16 ± 0.44	0.13 ± 0.38 ***
Serum total bilirubin (mg/dL)	0.73 ± 0.37	0.54 ± 0.26 ***	0.83 ± 0.34	0.67 ± 0.26 ***
Blood platelet (10^3^/µL)	259 ± 64.9	271 ± 63.6 ***	237 ± 54.1	262 ± 59.8 ***
GFR (mL/min)	77.7 ± 8.97	83.7 ± 15.7 ***	84.3 ± 14.8	120 ± 20.9 ***
Energy intake (EER%)	96.4 ± 32.3	104 ± 38.6 ***	90.8 ± 25.9	101 ± 32.9 ***
CHO intake (energy%)	69.1 ± 6.48	71.5 ± 6.77 ***	71.0 ± 7.03	71.8 ± 7.14 ***
Protein intake (energy%)	13.9 ± 2.29	13.6 ± 2.37 ***	13.4 ± 2.54	13.4 ± 2.57
Fat intake (energy%)	15.7 ± 4.98	13.8 ± 5.26 ***	14.5 ± 5.49	13.9 ± 5.56 ***

- No measurement in the cohort. SBP: Systemic blood pressure; DBP: Diastolic blood pressure; HDL: Serum high-density lipoprotein; CRP: Serum C-reactive protein; GFR: Glomerular filtration rate; HbA1C: Glycosylated hemoglobin; CHO, Carbohydrate; energy %, the percentage intake of energy. ** Significantly different from the men group in each cohort at *p* < 0.01, *** at *p* < 0.001.

**Table 2:** Accuracy of prediction models using the test set of the Ansan/Ansung cohorts.

Machine Learning Algorithm	Prediction of SMM			Prediction of FM		
	MSE ^a^	MAE ^b^	R² ^c^	MSE ^a^	MAE ^b^	R² ^c^
Linear regression	2.60	2.03	0.82	1.86	1.48	0.89
Support Vector Machines	2.71	2.12	0.80	1.98	1.52	0.87
XGBoost	2.56	2	0.82	1.82	1.43	0.89
Decision Tree	2.81	2.22	0.78	2.21	1.75	0.84
Random Forest	2.65	2.09	0.81	1.80	1.41	0.89
K-Nearest Neighbor (KNN)	3.08	2.4	0.74	2.16	1.68	0.85
Artificial neural network (ANN)	2.57	2	0.82	1.79	1.4	0.89

A prediction model was generated by training the results using 90% of Ansan/Asung cohort participants, and the accuracy of the prediction model was evaluated in the test set using mean square error (MSE) ^a^, mean-absolute-error (MAE) ^b^, and correlation efficiency of determination (R²) ^c^ to predict skeletal muscle mass (SMM) and fat mass (FM). Bold values were corresponding to the best algorithm.

**Table 3:** Adjusted means ^1^ and standard errors in anthropometric and biochemical parameters according to skeletal muscle mass (SMM) and fat mass (FM) in men at the urban hospital-based cohort.

Metabolic Parameters	HMLF (*n* = 4448)	HMHF (*n* = 3201)	LMLF (*n* = 2517)	LMHF (*n* = 231)
Predicted SMM (kg)	38.7 ± 0.03 ^b^	41.3 ± 0.05 ^a^	34.3 ± 0.03 ^d^	35.0 ± 0.06 ^c^
Predicted FM (%)	22.4 ± 0.03 ^c^	27.3 ± 0.04 ^a^	20.1 ± 0.06 ^d^	26.6 ± 0.15 ^b^
Body mass index (kg/m^2^)	24.1 ± 0.02 ^c^	27.0 ± 0.04 ^a^	21.8 ± 0.04 ^d^	24.6 ± 0.10 ^b^
Waist circumferences (cm)	84.7 ± 0.08 ^c^	92.5 ± 0.10 ^a^	78.3 ± 0.11 ^d^	85.4 ± 0.29 ^b^
Hip circumferences (cm)	95.6 ± 0.05 ^b^	100 ± 0.09 ^a^	89.2 ± 0.07 ^d^	91.8 ± 0.17 ^c^
Grip strength (kg)	39.9 ± 0.13 ^a^	38.6 ± 0.17 ^b^	36.3 ± 0.16 ^c^	33.8 ± 0.54 ^d^
Serum glucose (mg/dL)	98.6 ± 0.33 ^b^	102 ± 0.40 ^a^	98.6 ± 0.46 ^b^	102 ± 1.94 ^a^
Blood HBA1C (%)	5.70 ± 0.01 ^b^	5.93 ± 0.01 ^a^	5.72 ± 0.02 ^b^	5.91 ± 0.06 ^a^
Serum triglyceride (mg/dL)	141 ± 1.56 ^b^	177 ± 1.87 ^a^	124 ± 1.73 ^b^	173 ± 6.90 ^a^
Serum HDL (mg/dL)	50.2 ± 0.18 ^b^	46.9 ± 0.18 ^c^	54.3 ± 0.27 ^a^	49.2 ± 0.82 ^b^
GFR (mL/min)	84.0 ± 0.22 ^bc^	82.9 ± 0.27 ^c^	86.3 ± 0.29 ^a^	85.4 ± 0.93 ^ab^
Alcohol intake (g/day)	39.0 ± 1.09 ^a^	39.8 ± 1.06 ^a^	29.1 ± 0.77^b^	32.7 ± 3.24 ^b^

HBA1C: Glycosylated hemoglobin, GFR: estimated glomerular filtration rate. HDL: Serum high-density lipoprotein, HbA1C: Glycosylated hemoglobin, GFR: Estimated glomerular filtration rate. HMLF: High SSM and low fat-per, HMHF: High SSM and high fat-per, LMLF: Low SSM and low fat, LMHF: low SSM and high fat-per. The cutoff of SMM and fat mass was 48kg and 25%, respectively. ^1^ After adjusting for age, gender, residence area, education, and income status, and BMI. ^a,b,c,d^ Different superscript letters on the means in the same row indicated significant differences between the groups by the Duncan test.

**Table 4:** According to skeletal muscle mass (SMM) and fat mass (FM) in women at the urban hospital-based cohort, adjusted means and standard errors in anthropometric and biochemical parameters.

Metabolic parameters	HMLF (*n* = 5220)	HMHF (*n* = 10,522)	LMLF (*n* = 3615)	LMHF (*n* = 1368)
Predicted SMM (kg)	27.8 ± 0.02 ^b^	29.3 ± 0.02 ^a^	25.0 ± 0.02 ^d^	25.4 ± 0.02 ^c^
Predicted FM (%)	27.6 ± 0.03 ^c^	34.0 ± 0.03 ^a^	26.2 ± 0.04 ^d^	32.2 ± 0.06 ^b^
Body mass index (kg/m^2^)	21.8 ± 0.02 ^c^	25.5 ± 0.02 ^a^	20.6 ± 0.03 ^d^	23.4 ± 0.04 ^b^
Waist circumferences (cm)	74.3 ± 0.07 ^c^	82.6 ± 0.07 ^a^	69.4 ± 0.09 ^d^	76.6 ± 0.14 ^b^
Hip circumferences (cm)	91.3 ± 0.05 ^b^	96.4 ± 0.05 ^a^	85.9 ± 0.06 ^d^	89.04 ± 0.08 ^c^
Grip strength (kg)	24.6 ± 0.08 ^a^	23.3 ± 0.06 ^b^	22.6 ± 0.09 ^c^	20.5 ± 0.13 ^d^
Serum glucose (mg/dL)	90.8 ± 0.19 ^d^	95.4 ± 0.19 ^b^	92.5 ± 0.28 ^c^	96.4 ± 0.70 ^a^
Blood HBA1C (%)	5.52 ± 0.01 ^d^	5.76 ± 0.01 ^b^	5.61 ± 0.01 ^c^	5.85 ± 0.02 ^a^
Serum triglyceride (mg/dL)	92.3 ± 0.87 ^d^	125 ± 0.77 ^b^	104 ± 1.04 ^c^	137 ± 2.07 ^a^
Serum HDL (mg/dL)	60.3 ± 0.19 ^b^	55.1 ± 0.12 ^c^	61.2 ± 0.25 ^a^	55.8 ± 0.36 ^c^
GFR (mL/min)	122 ± 0.28 ^a^	120 ± 0.21 ^b^	118 ± 0.34 ^c^	116 ± 0.59 ^d^
Alcohol intake (g/day)	6.89 ± 0.21 ^a^	6.39 ± 0.41 ^a^	4.81 ± 0.23 ^b^	3.14 ± 0.32 ^c^

HBA1c: Glycosylated hemoglobin, GFR: Estimated glomerular filtration rate, HDL: Serum high-density lipoprotein. HMLF: High ASM and low fat-per, HMHF: High SSM and high FM, LMLF: Low SSM and low FM, LMHF: Low SSM and high FM. The cutoffs of SMM and FM were 36 kg and 30%, respectively. After adjusting for age, gender, residence area, education, and income status, and BMI. ^a,b,c,d^ Different superscript letters on the means in the same row indicated significant differences between the groups by the Duncan test.

## Data Availability

The data used in the present study are available on request from the corresponding author.

## Supplementary Materials

The following are available online at https://www.mdpi.com/article/10.3390/jcm10102133/s1, Figure S1: Age distribution of Ansan/Ansung and city hospital-based cohorts.
