# Supplementary material for: An Inverse Relation between Hyperglycemia and Skeletal Muscle Mass Predicted by Using a Machine Learning Approach in Middle-Aged and Older Adults in Large Cohorts
Source: J Clin Med. 2021 May 14;10(10):2133. doi: 10.3390/jcm10102133 (PMC8156777; doi:10.3390/jcm10102133)

Supplemental figure S1: Age distribution of Ansan/Ansung and city hospital-based cohorts.

A. Ansan/Ansung cohort

B. City hospital-based cohort

C. Train and test sets in Ansan/Ansung cohort for predicting skeletal muscle mass

D. Train and test sets in Ansan/Ansung cohort for predicting fat mass

A.

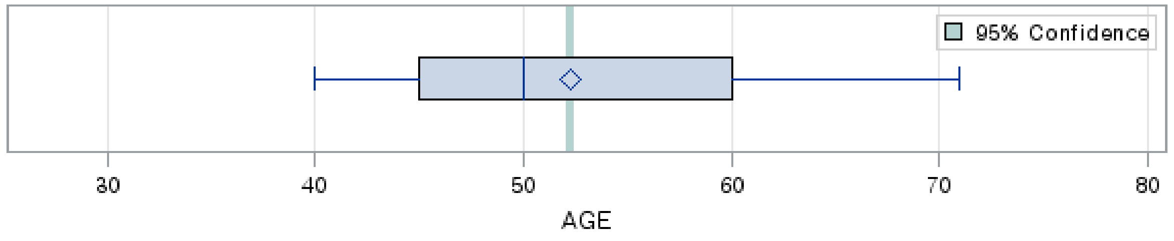

B.

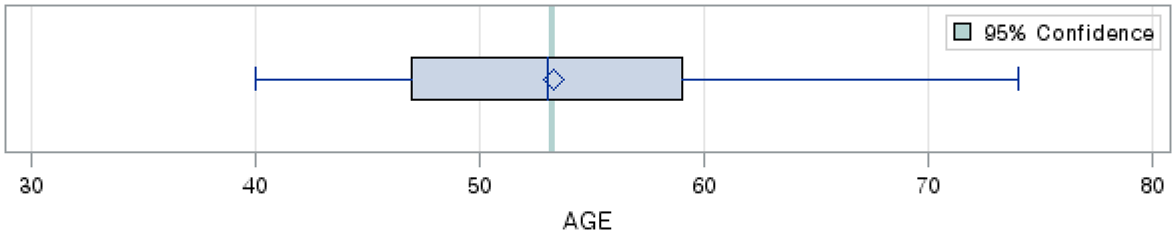

C.

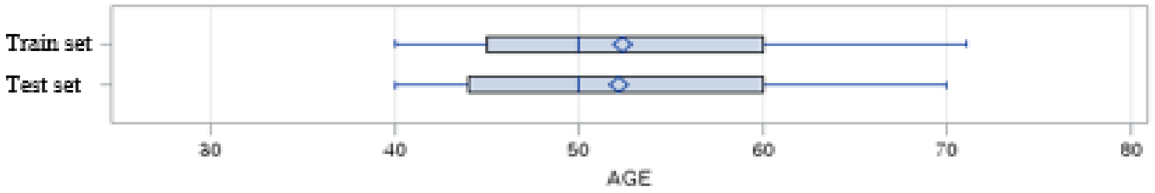

D.

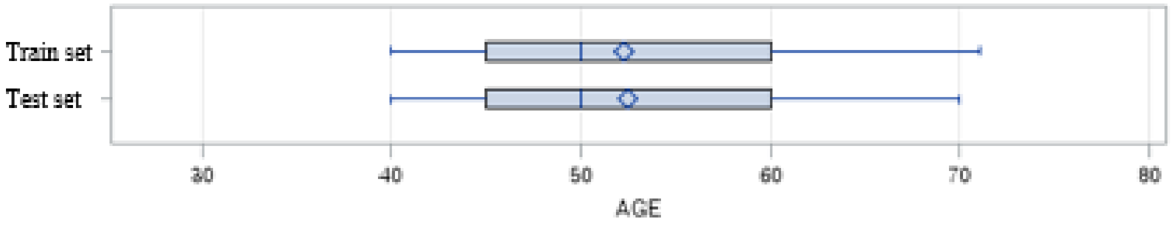

Supplement: Supplementary file 1 [file jcm-10-02133-s001.zip › jcm-1143546-supplementary.pdf]
